# Does Medicine without Evolution Make Sense?

**DOI:** 10.1371/journal.pbio.0050112

**Published:** 2007-04-17

**Authors:** Catriona J MacCallum

## Abstract

Should evolutionary biology contribute to the education of medical students?

It is curious that Charles Darwin, perhaps medicine's most famous dropout, provided the impetus for a subject that figures so rarely in medical education. Indeed, even the iconic textbook example of evolution—antibiotic resistance—is rarely described as “evolution” in relevant papers published in medical journals [1]. Despite potentially valid reasons for this oversight (e.g., that authors of papers in medical journals would regard the term as too general), it propagates into the popular press when those papers are reported on, feeding the wider perception of evolution's irrelevance in general, and to medicine in particular [1]. Yet an understanding of how natural selection shapes vulnerability to disease can provide fundamental insights into medicine and health and is no less relevant than an understanding of physiology or biochemistry.

One reason that evolution doesn't figure prominently in the medical community is that although it makes sense to have evolution taught as part of medicine, that doesn't make it essential. As explained at a meeting on evolution and medicine I recently attended in York, United Kingdom (the Society for the Study of Human Biology and the Biosocial Society's 2006 symposium, “Medicine and Evolution”), medicine is primarily focused on problem-solving and proximate causation, and ultimate explanations can seem irrelevant to clinical practice. Crudely put, does a mechanic need to understand the origins, history, and technological advances that have gone into the modern motor vehicle in order to fix it?

Randolph Nesse (University of Michigan) and colleagues think otherwise [2], and have been campaigning for evolution to be recognized and taught as a basic science to all medical students (see also the Evolution and Medicine Network, http://www.evolutionandmedicine.org). It has been more than 10 years since he and George Williams published their classic book *Why We Get Sick: The New Science of Darwinian Medicine* [3]. Other landmark texts linking evolution to health have been written since then, with new editions on the way [4–6], and the research field is blossoming. Still, as Nesse mentioned at the start of the York meeting, there are only a handful of medical schools in the United States and in the United Kingdom with an evolutionary biologist listed as such on the faculty.

The most obvious examples of evolutionary biology's importance to medical understanding are related to infectious disease [7]. As Jon Laman (Erasmus University, The Netherlands) pointed out at the meeting, the immune system provides the perfect platform to explain the medical relevance of the exquisite evolutionary relationships between pathogens and their hosts. Understanding how virulence evolves, for example, can help predict the potential, sometimes counterintuitive (and controversial) negative consequences of imperfect vaccination [8,9]. But evolution can also tell us that the origin of HIV was precipitated by a jump across the primate species barrier [10] and enables us to predict the imminent arrival of avian flu and the mutations most likely to be responsible for that evolutionary leap from birds to humans [11]. Where epidemiological and population genetic processes occur on the same time scale, the emerging field of “phylodyamics” can also inform us about the timing and progression of pathogen adaptation more generally [12].

The relevance of evolution to medicine is, however, much broader. Participants at the York meeting discussed not only how vulnerability to cancer is an inevitable but unfortunate consequence of imperfect human engineering and natural selection (Mel Greaves, Institute of Cancer Research, UK), but how life history theory can potentially explain patterns of pregnancy loss (Virginia Vitzthum, Indiana University), how a comparative approach applied to different human cultures and different primates can improve rates of breastfeeding (Helen Ball, University of Durham), whether clinical depression has an adaptive origin (Lewis Wolpert, University College London), and if suicide attempts are really just evolutionary bargaining chips in intense social disputes (Ed Hagen, Humboldt University).

As with any emerging field, ideas change and the science is challenged. The thrifty gene concept [13]—that some populations (e.g., from Polynesia) are particularly susceptible to type 2 diabetes and heart disease because of past selection pressure specifically during times of famine—no longer enjoys the support it once had [14]. Tessa Pollard (University of Durham, UK) explained that the so-called Syndrome X is now considered to be the result of more general exposure to a rapid change in lifestyle as Western society encroached on these populations during the mid-20th century. The relationship between changing environment, diet, and susceptibility to disease, however, is also far from clear. Many diet-related conditions that typify industrialized populations—e.g., obesity, hypertension, and tooth decay—have been explained as resulting from an evolutionary mismatch between our over-refined, fat-filled contemporary diet and the environment to which humans were once ideally adapted. Sarah Elton (Hull York Medical School, UK) cautioned that while this analogy (the “environment of evolutionary adaptedness”) has been useful as a research tool and has led to public health campaigns for better diets (more seeds, nuts, fish oil, etc.), recreating such a typical “Stone Age diet” as a benchmark can be misleading. Human ecology in the past was at least as variable as human (and other primate) ecology is today.

Surprisingly, an evolutionary framework to study human variation can be seen as counterproductive. George Ellison (St. George's Medical School, UK) provided an example, although not concerning evolutionary medicine, about a statistically flawed study leading to spurious conclusions about regional variation in IQ (which I won't promulgate here). However, bad papers are published in all subjects and are a failure of scientists and the peer-review system, not the science. These should not provide an excuse to dismiss the relevance of evolution to medicine (or to any other life science). Even at a very basic level, medical students can draw insights from evolution they cannot obtain from other core sciences on their course. Paul O'Higgins (Hull York Medical School) noted that it is much easier for medics to learn the nerves involved in the brachial plexus (the nerves supplying the arm) if they first understand the origin of the pentadactyl limb.

It is not the case, however, that all clinicians fail to see the relevance of evolution. Gillian Bentley (now at University of Durham) conducted a series of interviews with leading biologists and clinicians when she was based at Imperial College London. What was surprising was not the positive endorsement of evolution by the geneticists and evolutionary biologists but the enthusiasm of practicing medical doctors for the topic, whether involved in the active birth movement or dealing with major trauma in intensive care. Indeed, several local clinicians attended the York meeting and helped lead the discussions.

Ironically, the hardest task in adding evolutionary/Darwinian medicine to medical curricula may well be soliciting support from medical students. Although Paul O'Higgins thought a comparison of the brachial plexus to the pentadactyl limb was helpful, not all his students agreed—complaints were lodged that he was forcing evolution on them. That lack of support was also reflected in the participation of only three medical students at the York meeting (albeit enthusiastic ones), despite being widely publicized. It is not clear whether this is because medical students are more overburdened than most or because of a more deep-rooted resistance to the subject, reflecting wider political and religious prejudice against evolution. But evolutionary medicine isn't and shouldn't be controversial, and the best way to challenge prejudice is through education. As the oft-quoted Theodosius Dobzhansky wrote in 1973, “Nothing in biology makes sense except in the light of evolution” [15]. The time has clearly come for medicine to explicitly integrate evolutionary biology into its theoretical and practical underpinnings The medical students of Charles Darwin's day did not have the advantage of such a powerful framework to inform their thinking; we shouldn't deprive today's budding medical talent of the potential insights to be gained at the intersection of these two great disciplines.

## 

**Figure:**
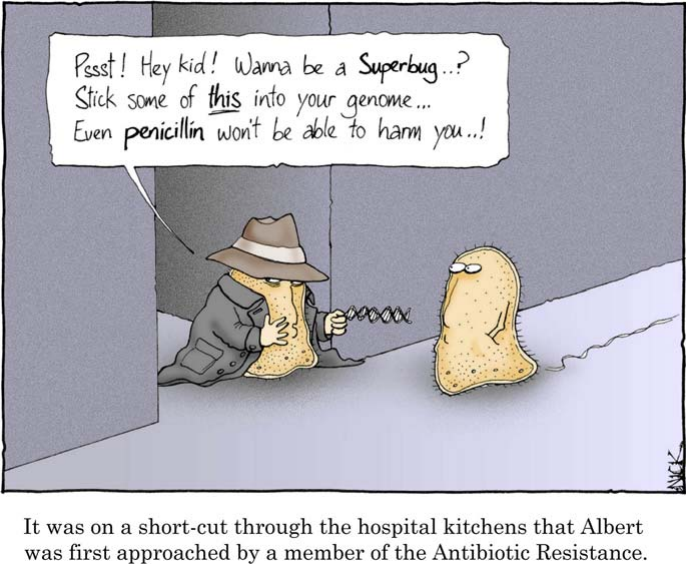
It was on a short-cut through the hospital kitchens that Albert was first approached by a member of the Antibiotic Resistance. Image: Nick D. Kim
